# A new approach to mentoring for research careers: the National Research Mentoring Network

**DOI:** 10.1186/s12919-017-0083-8

**Published:** 2017-12-04

**Authors:** Christine A. Sorkness, Christine Pfund, Elizabeth O. Ofili, Kolawole S. Okuyemi, Jamboor K. Vishwanatha, on behalf of the NRMN team, Maria Elena Zavala, Theresa Pesavento, Mary Fernandez, Anthony Tissera, Alp Deveci, Damaris Javier, Alexis Short, Paige Cooper, Harlan Jones, Spero Manson, Dedra Buchwald, Kristin Eide, Andrea Gouldy, Erin Kelly, Nicole Langford, Richard McGee, Clifford Steer, Thad Unold, Anne Marie Weber-Main, Adriana Báez, Jonathan Stiles, Priscilla Pemu, Winston Thompson, Judith Gwathmey, Kimberly Lawson, Japera Johnson, Meldra Hall, Douglas Paulsen, Mona Fouad, Ann Smith, Rafael Luna, Donald Wilson, Greg Adelsberger, Drew Simenson, Abby Cook, Monica Feliu‐Mojer, Eileen Harwood, Amy Jones, Janet Branchaw, Stephen Thomas, Amanda Butz, Angela Byars‐Winston, Stephanie House, Melissa McDaniels, Sandra Quinn, Jenna Rogers, Kim Spencer, Emily Utzerath, DUPLICATE of Weber-Main, Veronica Womack

**Affiliations:** 1 Institute for Clinical and Translational Research, University of Wisconsin, Madison, WI 53705 USA; 2 Department of Medicine, Morehouse School of Medicine, Atlanta, GA 30310 USA; 3 Department of Family and Preventive Medicine, University of Utah School of Medicine, Salt Lake City, UT 84108 USA; 4 Center for Diversity and International Programs, University of North Texas Health Science Center, Fort Worth, TX 76107 USA

## Abstract

**Background and purpose:**

Effective mentorship is critical to the success of early stage investigators, and has been linked to enhanced mentee productivity, self-efficacy, and career satisfaction. The mission of the National Research Mentoring Network (NRMN) is to provide all trainees across the biomedical, behavioral, clinical, and social sciences with evidence-based mentorship and professional development programming that emphasizes the benefits and challenges of diversity, inclusivity, and culture within mentoring relationships, and more broadly the research workforce. The purpose of this paper is to describe the structure and activities of NRMN.

**Key highlights:**

NRMN serves as a national training hub for mentors and mentees striving to improve their relationships by better aligning expectations, promoting professional development, maintaining effective communication, addressing equity and inclusion, assessing understanding, fostering independence, and cultivating ethical behavior. Training is offered in-person at institutions, regional training, or national meetings, as well as via synchronous and asynchronous platforms; the growing training demand is being met by a cadre of NRMN Master Facilitators. NRMN offers career stage-focused coaching models for grant writing, and other professional development programs. NRMN partners with diverse stakeholders from the NIH-sponsored Diversity Program Consortium (DPC), as well as organizations outside the DPC to work synergistically towards common diversity goals. NRMN offers a virtual portal to the Network and all NRMN program offerings for mentees and mentors across career development stages. NRMNet provides access to a wide array of mentoring experiences and resources including MyNRMN, Guided Virtual Mentorship Program, news, training calendar, videos, and workshops. National scale and sustainability are being addressed by NRMN “Coaches-in-Training” offerings for more senior researchers to implement coaching models across the nation. “Shark Tanks” provide intensive review and coaching for early career health disparities investigators, focusing on grant writing for graduate students, postdoctoral trainees, and junior faculty.

**Implications:**

Partners from diverse perspectives are building the national capacity and sparking the institutional changes necessary to truly diversify and transform the biomedical research workforce. NRMN works to leverage resources towards the goals of sustainability, scalability, and expanded reach.

## Background

Robust mentorship has been linked to enhanced mentee productivity, self-efficacy, and career satisfaction; it is also an important predictor of the success of researchers in training [1–12]. Unfortunately, studies have reported that trainees from minority groups typically receive less mentoring than their non-minority peers [13–16]. In studies on barriers to National Institutes of Health (NIH) funding, minority investigators indicated that inadequate mentoring, lack of understanding about institutional requirements, lack of institutional support, and social, cultural, and environmental factors all posed obstacles to success [17]. While a lack of mentoring is not unique to underrepresented investigators, the effect disproportionately impacts those from underrepresented groups, especially those in majority/white institutions. A follow-up article to the Ginther report that examined possible reasons for the disparity in NIH funding also hypothesized that variability in access to mentoring may be one of the contributing factors [18]. Mentoring programs must therefore address these concerns and create a supportive web of invested mentors and peers who are committed to advancing the careers of investigators from traditionally underrepresented groups.

NIH leadership has directly addressed the science of diversity, citing the racial, ethnic, gender, and economic imbalance of the US biomedical research workforce as limiting the promise of building knowledge and improving the nation’s health. Drs. Hannah Valentine and Francis Collins have argued that recruitment and retention of a diverse set of minds and approaches is vital to harnessing the complete intellectual capital of the US [19]. NRMN has purposefully adopted the NIH Diversity Statement [20] to guide its inclusion of and invitation to underrepresented populations to access its networking, mentoring, and career development services and programs. The NIH has initiated the Diversity Program Consortium (DPC), a national collaborative to work together with institutions to advance the overarching goal of developing, implementing, assessing and disseminating innovative, effective approaches to research training and mentoring. The DPC consists of three integrated initiatives in response to the cited diversity imbalance: Building Infrastructure Leading to Diversity (BUILD), the Coordination and Evaluation Center (CEC), and the National Research Mentoring Network (NRMN).

We offer the NRMN approach as a template to other comparable international institutes of health research, such as the National Institute of Health Research United Kingdom (NIHR UK) [21], the Canadian Institutes of Health Research CIHR) [22], and the Australian Institute of Health and Welfare (AIHW) [23]. Each of these institutes share a mission of creating new scientific knowledge and enabling translation into improved health, more effective health services and products, and strengthening national health care systems. Further, these kindred institutes provide leadership and support to the next generation of researchers, leaders, and professionals across their countries. Whereas, the challenges may not be identical to the US, these institutes each embrace workforce diversity programs, and have targeted attraction of talent across the same underrepresented groups as the US, but also unique indigenous populations, aboriginal peoples, and immigrants. Thus, the NRMN approach and framework is very relevant to others with similar missions.

The purpose of this manuscript is to outline the mission, goals, infrastructure, and programs developed by the NRMN to address the barriers outlined by the Ginther report [18] and significantly contribute to national efforts to enhance the size, quality, diversity, and productivity of the biomedical research workforce trained to improve population health. NRMN is a nationwide consortium of biomedical professionals and collaborating institutions, working to provide all trainees across the biomedical, behavioral, clinical and social sciences with evidence-based mentorship and professional development programming that emphasizes the benefits and challenges of diversity, inclusivity, and culture within mentoring relationships, and more broadly the research workforce.

NRMN is a cooperative agreement between the National Institute of General Medical Sciences and five academic institutions (Boston College, Morehouse School of Medicine, University of Utah-Salt Lake City, University of North Texas Health Sciences Center, and University of Wisconsin-Madison). This Network is led by a leadership team, directing Administrative, Mentorship and Networking, Mentor Training, Professional Development, and Research Recruitment and Outreach Cores. Cores are working groups of researchers tasked with specific functions. This leadership team is joined by a diverse and synergistic set of associate/assistant directors, co-investigators, consultants, and Core staff. NRMN partners with diverse stakeholders from the NIH DPC, including BUILD and non-BUILD institutions as well as NRMN supplement and pilot grant awardees to work synergistically, guided by an integrated logic model of program components and goals. Whereas, NRMN has Core-specific aims, operational infrastructure, functions, and associated budgets consistent with the requirements of the original NIH application request, the overarching goal is to implement cross-Core initiatives, as outlined in this manuscript.

### The opportunity

Despite evidence of mentoring’s importance, little is known about the complexities of mentoring relationships both in terms of how individual cultural differences like race, ethnicity, and gender influence these relationships as well as the effectiveness of different forms of mentoring. Moreover, it still remains unclear which types of mentoring relationships (e.g., dyads, dual or multi-mentored, peer) and which modes of mentoring (e.g., formal, informal, face to face, online, short-term, long-term) have the greatest impact on mentee success, conditioned by contexts and career stage. NRMN offers the opportunity to address the lack of training and standardization in effective mentoring methods, and to expand research on mentoring and identify some best practices*.* Along these lines, investigators within NRMN are working to refine aligned mentor and mentee standards, and their accompanying metrics, for effective mentoring relationships at various career stages using attributes predicted to increase the number (and diversity) of those who enter, persist in, and launch successful biomedical science careers. Two recent manuscripts provide detailed definitions of mentoring being applied in NRMN and a list of proposed attributes and metrics for effective mentoring relationships [24, 25]. These definitions and attributes have guided NRMN program development described below.

To capitalize on the opportunity NRMN presents for improving mentoring relationships and their impact, as well as to foster research in this area, NRMN was designed with a theoretical frame in mind described in detail in Pfund et al. 2016 [24]. Importantly, NRMN activities: (a) are grounded in a robust conceptual model of persistence focused on social cognitive career theory and the formation (and reformation) of science and cultural identity across developmental stages; (b) authentically address bias, stereotype threat, and cultural ignorance; (c) focus on the formal preparation of both mentors and mentees; (d) are built upon process-based, community-building approaches to mentor and mentee training; and (e) include established multimodal training formats and proven train-the-trainer efforts that allow for rapid scale-up and sustainability.

### NRMN program overview

**Fig. 1:**
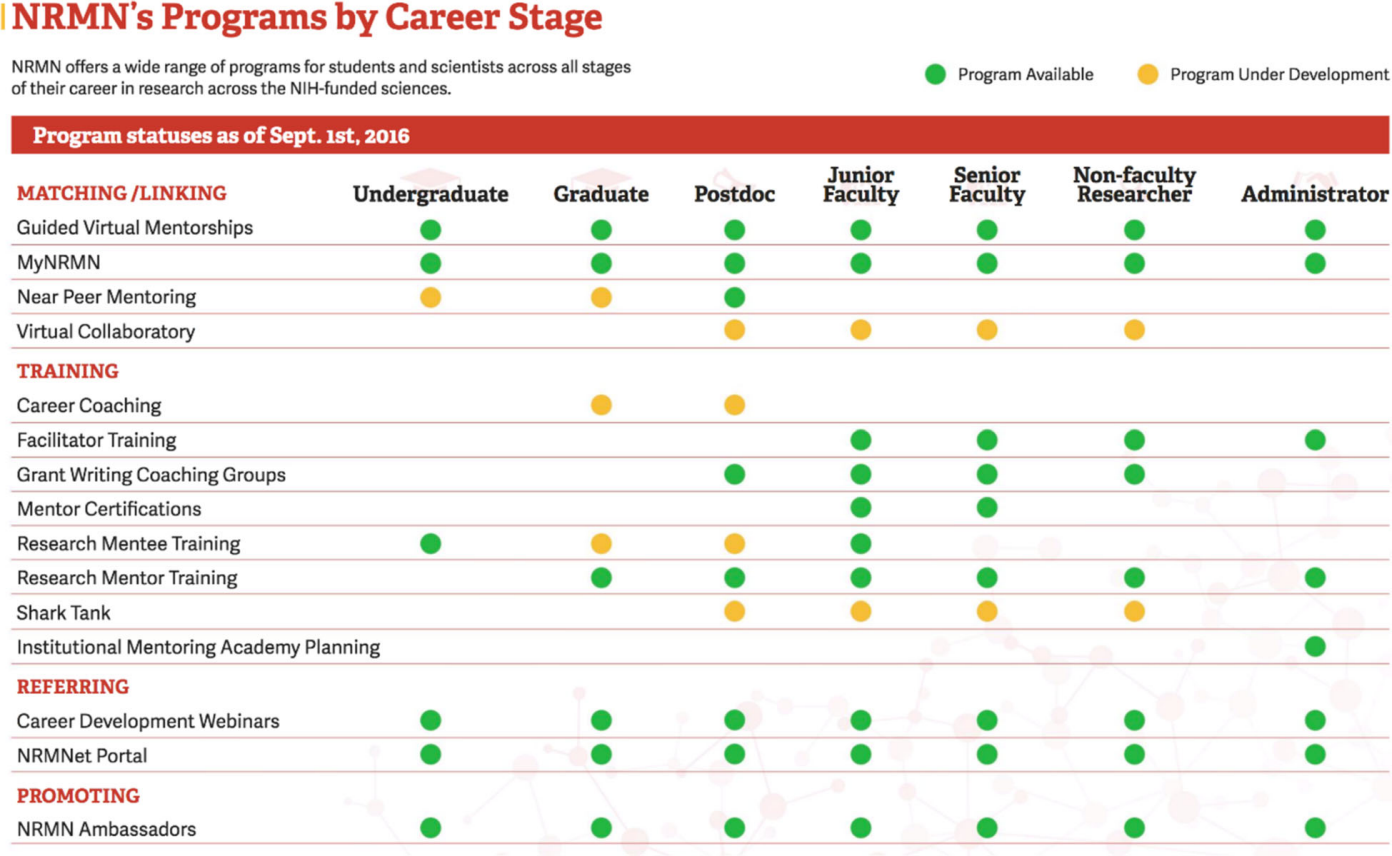
NRMN Programs by Career Stage, across the Four Program Goals

NRMN strives to employ and disseminate best practices for mentorship training, with unique opportunities for networking and professional development intended to facilitate the attainment of hallmarks of successful research career progression for mentees at each career stage. The NRMN program offerings (see Fig. 1) were specifically designed to achieve four goals: 1) increase access to mentoring across career stages; 2) improve mentoring relationships, broadly defined, through training; 3) increase access to research resources and career development and 4) promote the value of mentoring. The expected outcomes and short- and long- term outcomes for each of these four programmatic areas are part of an NRMN Integrated Logic Model, as described by Guererro et al. in this issue [26]. NRMN programs designed to achieve each goal are highlighted below.

### Evaluation of NRMN activities and outcomes

NRMN is interested in both program-specific formative evaluation and cross-program evaluation of participant outcomes. A NRMN-wide evaluation team has developed comprehensive NRMN-wide monitoring and evaluating tasks, eliminating redundancies in data collection, management, and reporting and using well-documented evaluation processes and tools that are simple, flexible, useful, feasible, and accessible. This evaluation team is responsible for building, and overseeing the monitoring and evaluation capacity across NRMN so data can be used to monitor NRMN program progress, assess short-term achievements, and contribute to the tracking of long-term outcomes. This team develops evaluation tools and processes, and is charged with building mentoring and evaluation capacity inside the NRMN organization by empowering staff to own data created and used for monitoring progress and assessing and disseminating achievements. Importantly, the NRMN evaluation team collaborates with the DPC CEC, which is responsible for intermediate and long-term participant and workforce outcome evaluation. A detailed description of NRMN evaluation in this collaborative context can be found in the article by Guererro et al. in this issue [26].

### NRMNet web portal

**Fig. 2:**
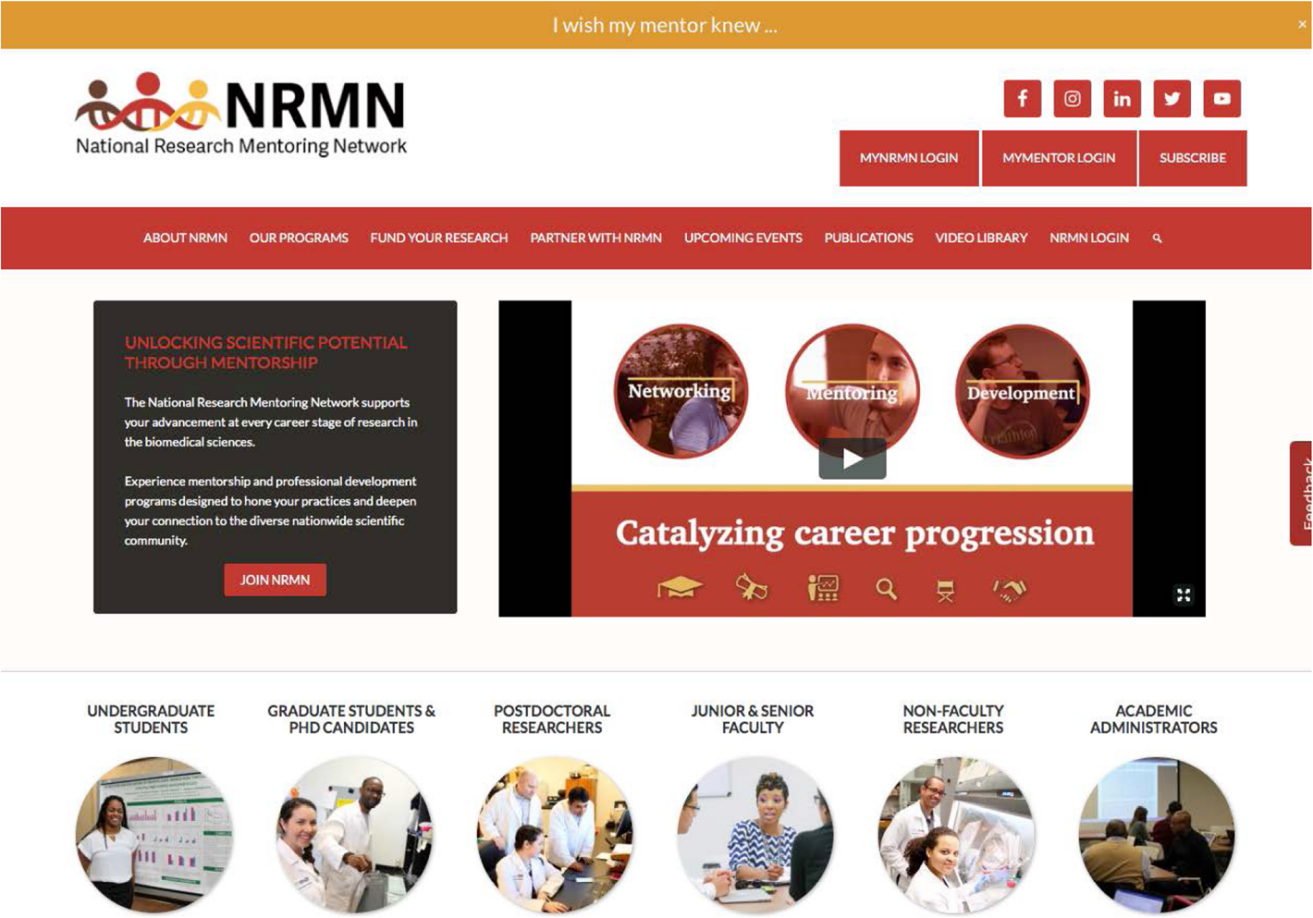
Snapshot of the NRMNet Website; figure designed by the authors in response to permission. (The authors have received consent to publish from those in the photos)

The NRMNet web portal (www.nrmnet.net) is the gateway to career stage-specific resources that support every end user, from undergraduates to academic administrators. Figure 2 is a snapshot of the NRMNet website. Users can access mentorship and professional development programs designed to hone their skills and deepen connections to the diverse nationwide scientific community. The platform also includes an events calendar, latest videos specific to career development and social media, and other offerings, which users can view for free.

**Fig. 3:**
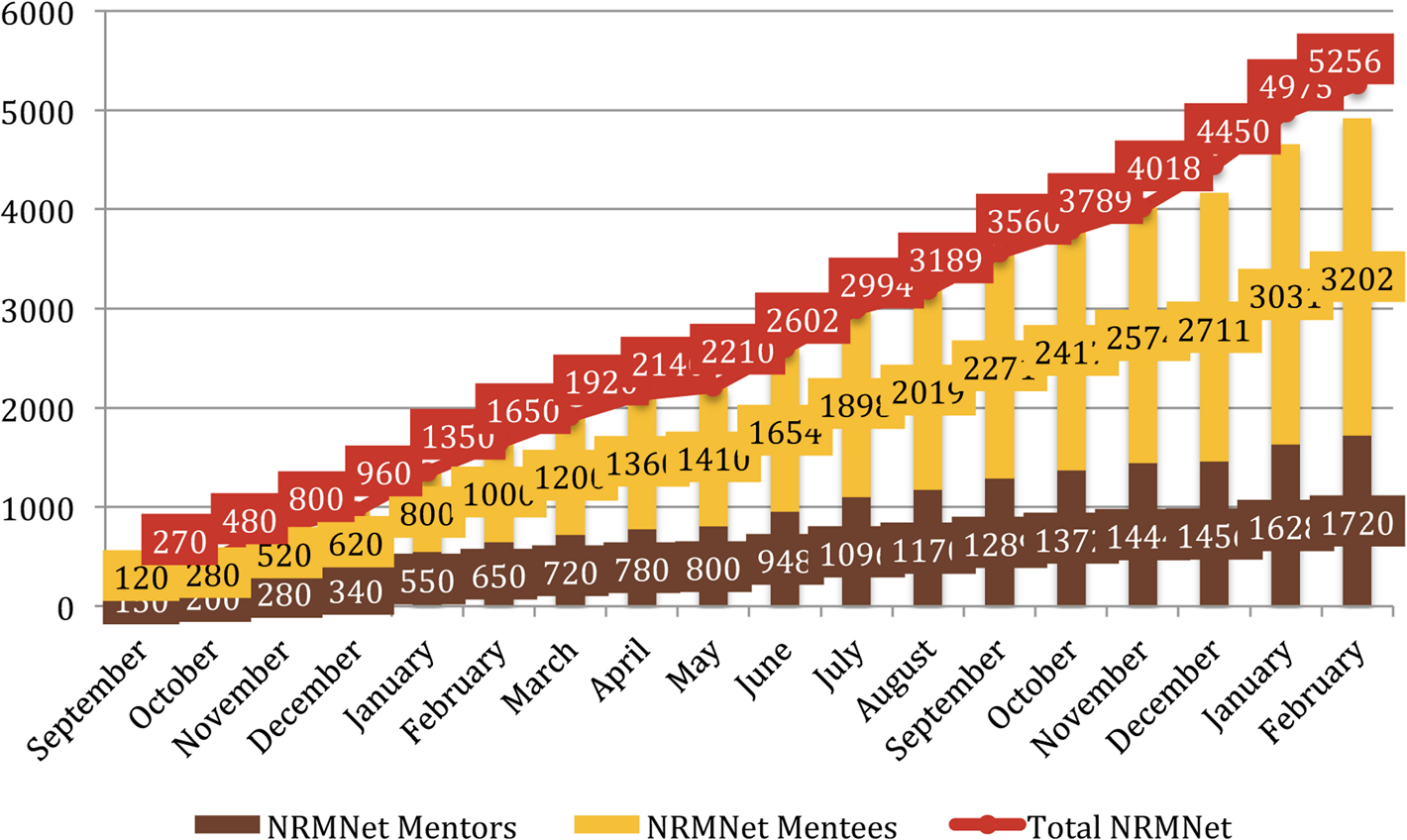
NRMNet Participant Growth via the Web Portal (Sept. 2016-Feb. 2017). Totals in December 2016, January 2017, and February 2017 contain incomplete profiles for which a designation of either “mentor” or “mentee” was not yet specified, hence the apparent gap between the combined mentor and mentee totals relative to Total NRMNet count in those months

Google Analytics tracks NRMNet website traffic and registration on the NRMN portal. During 2016, the NRMN web portal was visited by an average of 2500 unique users monthly (59% as new visitors and 41% returning). Figure 3 illustrates the growing numbers of NRMNet web portal registrants during the initial years of the program. Importantly, this is an underestimate as not all program participants were able to register through the website as the program launched. Of those tracked through 2016, 64% of the currently registered mentees are from underrepresented groups and 68.5% are female. Forty-two percent of mentors self-identify as an underrepresented group and 55.8% are female. NRMNet registrants increased by 45% from 2015 to 2016, and represent 50 states and Puerto Rico.

### Programs developed to achieve NRMN goals

#### Goal 1. Matching and linking: Increasing access to mentoring across career stages

**Fig. 4:**
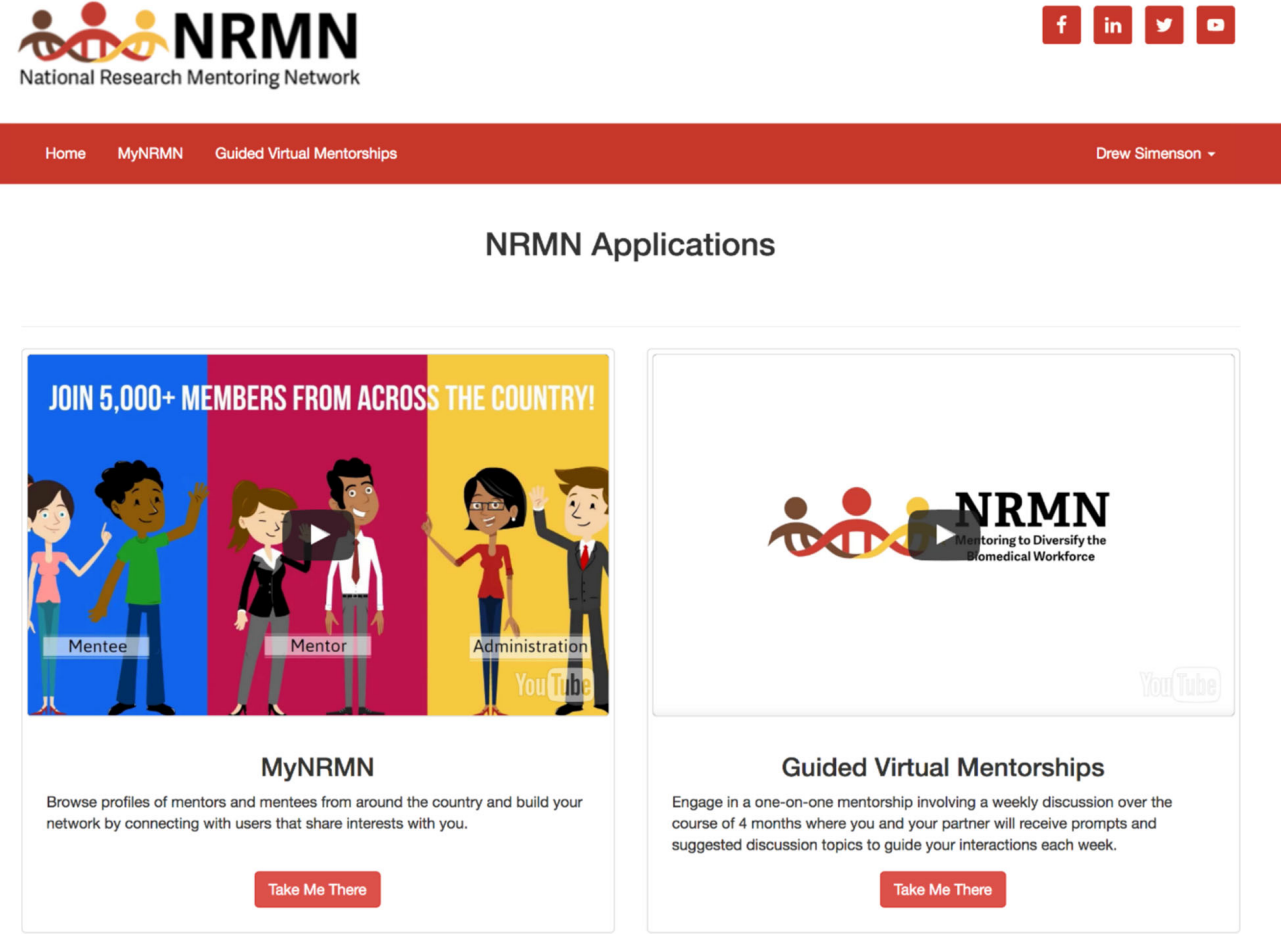
Snapshot of NRMNet Portal Homepage, Featuring the MyNRMN and Guided Virtual Mentorships Applications

In order to increase the access of diverse mentees to mentors across the country, NRMN has created multiple programs to link highly knowledgeable and skilled mentors from various disciplines with talented, motivated and diverse mentees from the undergraduate student to early career faculty level. Two of these mentoring and networking applications are MyNRMN and the Guided Virtual Mentorship Program which can be accessed by a registered user on NRMNet web portal. Figure 4 is a snapshot of the homepage, featuring these applications.

#### MyNRMN

MyNRMN is a powerful social networking platform designed to help mentees and mentors to connect professionally. Participants can browse profiles of mentors and mentees and build their network by connecting with users who share interests with them. The user can utilize the dashboard to chat in real time with others in their network, send messages seeking advice, share documents, build their resume with the CV Builder tool, and schedule appointments to collaborate with others using a personalized calendar. MyGroups, a MyNRMN feature released in December 2016, allows for members to participate in collaborative activities like group chats, and to share documents and images within an area of interest. The group types include Public, Private (only for those invited), Invite Only (accessed solely by owners and members limited to owner request to join), and Announcements.

#### MyMentor: Guided virtual mentorship program

Mentees looking for a more structured interaction with a mentor can sign up for the guided virtual mentorship program. Participants engage in a one-on-one, four-month long virtual engagement involving weekly discussions where the mentee and mentor receive prompts and suggested discussion topics to guide their interactions. Once the four-month mentorship is complete, mentees can select a new mentor and gain the perspectives of multiple mentors over time, or continue to interact with the same mentor by inviting them to engage in another mentorship through Guided Virtual Mentorship or through the MyNRMN networking program. There are over 70 discussion topics for different developmental levels (undergraduate student through postdoctoral trainee) on the platform. Over 280 matches are in process or have occurred to date with continued growth.

#### Goal 2. Training: Improving mentoring relationships, broadly defined, through training

NRMN is developing, enhancing, implementing and evaluating career-stage appropriate training for mentors and mentees with a focus on broadening and deepening impact of mentoring relationships on the persistence and success of diverse biomedical researchers. This plan includes training for 1) research mentors and mentees, 2) career coaches and trainee coaching groups, and 3) grantsmanship coaches and trainees preparing to write or in the process of writing proposals. Training is implemented in a variety of modes including in-person training at institutions, regional hubs, and disciplinary/ professional society meetings, in addition to synchronous and asynchronous online training. Training materials are largely based on previously developed and tested approaches. Longitudinal evaluation data are being collected from all training events, in partnership with the national CEC [26] and include: participation; satisfaction with elements of training; self-reported knowledge and skill gains from training; and intent to apply/ actual application of knowledge and skills gained from training. Finally, NRMN is working to improve these programs based on evaluation data, augment them, and disseminate the evidence-based models in ways that are sustainable. Each type of NRMN training is described below.

#### Research mentor and mentee training

Face-to-face training for mentors and mentees engaged in traditional research relationships, such as those between graduate students and their PhD advisors, is offered through NRMN. These trainings are based, in part on the evidence-based curricula series, *Entering Mentoring* [27–30] and *Entering Research* [31, 32] and focus on six targeted competencies. New NRMN training modules are also being developed to address target areas such as cultural awareness, acknowledging that even when mentors are available to mentees from underrepresented groups, mentoring strategies and structures are often insufficient to cope with the barriers they face [33]. Some of these challenges include marginalization, overt and covert racism, and disproportionate involvement in activities that do not advance careers (e.g. serving on numerous committees seeking minority representation, engaging in many community outreach programs) [14–16]. A six-hour module for culturally aware mentorship (CAM) was designed and beta-tested by NRMN researchers to help participants examine their own racial and ethnic identify, and use insights from these reflections to identify their personal assumptions, biases, and privileges that may impact their research mentoring relationships. Mentor participants reported significant skill gains across eight skills associated with culturally aware mentoring (e.g., “Thinking about how the research experience might differ for mentees from different racial/ethnic groups”), with an average skill gain of 1 point on a 7-point scale.

During just the first 2 years, NRMN implemented 72 training events for 1427 mentors and collected evaluation data to assess training across diverse mentors varying in background, career stage, discipline, context, and dosage of training. Satisfaction ratings across all trainings were high. Knowledge and skill gains were reported across all six competencies on the validated Mentoring Competency Assessment [34], In some cases, mentors who participated in a six-hour training rated the quality of their mentoring a full point higher post-training with a mean of 4.36 (before) and 5.38 (after) on a 7-point scale. Combined data across trainings, have generated a rich source of data that will allow NRMN investigators to determine robust outcomes from mentor training across a wide range of variables and inform best practices for dissemination.

##### Asynchronous online mentor training

*Optimizing the Practice of Mentoring* (OPM) is an asynchronous, self-paced online course for senior faculty mentors of junior faculty, postdoctoral scholars, and graduate student mentees in biomedical, clinical, and translational science disciplines. Learners engage with material on their own time, at their own pace, and with different levels of engagement, with users averaging 90 min to complete the course. Content is organized into five modules that cover mentoring models, mentor roles and responsibilities, structure and dynamics of the mentoring relationship, and strategies for facilitating and addressing challenge to the mentoring process. Evaluation data are being collected post-training for OPM as a standalone module and in combination with other mentor training programs. As an example, among OPM completers, 92% expressed intention or possible intention of making changes to their mentoring practices. Currently, an NRMN research group is adapting the OPM course for the mentors of undergraduates.

##### Synchronous online mentor training

NRMN offers mentor training on an online environment where participants and instructors meet weekly for 2 h, communicating via audio and video conferencing. This online environment includes a host of functions that promote rich interactions, including an interactive electronic whiteboard, built-in chat functions that harness the power of parallel processing, and even breakout rooms that enable smaller group discussions. In spring 2016, two sections of the synchronous online mentor training were offered, based on the evidence-based approach implemented through the University of Wisconsin Center for the Integration of Research, Teaching and Learning (UW CIRTL [35] with priority given to mentors of undergraduate students at BUILD institutions. Evaluation data from these six 2-h sessions, covering all of the competencies in *Entering Mentoring* in addition to new modules, indicated overall satisfaction with the online training experience and learning gains in the targeted mentoring competencies.

##### Mentee training

During the first 2 years of NRMN, much focus was on development of new and adapted materials for training mentees across career stages. An NRMN subgroup of 21 individuals from nine institutions is actively revising the original evidence- based *Entering Research* curriculum for undergraduates [31, 32]. This subgroup is developing and adding new activities and assignments based on NRMN priorities, including cultural awareness, science identity, and research self-efficacy. Through activities, case studies, and discussion, mentees who participate in Entering Research-based trainings are introduced to important topics and concepts organized around five key areas of trainee development: research skills; interpersonal skills; psychosocial skills; diversity/culturally focused skills; and professional and career development skills. The revised Entering Research curriculum is designed to be fully customizable based on the needs of individual institutions and their mentees. To date, 83 activities have been developed or adapted and are being peer reviewed by members of the subgroup. The revised undergraduate curriculum will be tested with several partners, including several BUILD institutions. The revised graduate student activities will also be tested. The subgroup will also determine whether the curricular themes transfer to other career stages and identify additional themes relevant to more advanced career stages.

##### Career coaching

To augment classic research mentoring, NRMN has launched an evidence-based career coaching initiative, focused on providing trainees with the skills and support they need to effectively navigate their career choices and pathway(s) [25, 36]. Career coaching can augment and compliment research mentoring by providing guidance outside the traditional research relationship. Coaches can offer confidential guidance, scientific expertise, psychosocial support, and career advice. This coaches’ training and training for graduate students launched initially for the American Society for Pharmacology and Experimental Therapeutics (ASPET) in 2016. Four coaches, each with a coaching group of four PhD students and two postdoctoral trainees, participated in a half-day coach training ahead of a daylong coaching group kickoff. Coaching continues virtually for 12 months thereafter. The annual training incorporates some elements of the culturally aware mentorship module noted above. Data from coaches’ training are used to inform expansion of this effort.

##### Coaching for improved Grantsmanship

NRMN has implemented and is testing four unique but complimentary coaching models specific for grant writing, tailored to the level of experience and readiness of the trainees. Programs are structured to engage either less or more experienced post-doctoral associates or junior faculty, and have various schedules and timelines. To achieve scalability, all programs recruit highly successful “Coaches-in-Training” to learn approaches and group facilitation skills.

NRMN’s grant writing coaching models for postdoctoral fellows and junior faculty fall under two categories. First, models for those who will be working on a grant proposal sometime within the next year, which combine on-site grant writing and professional development experiences with distance learning techniques that include online digital meetings. Content begins with writing skills and grant proposal basics such as developing a research question. The Coach or Advisor helps mentor those in the program. The second model is designed for those currently working on an in-progress grant proposal. This model begins with an intensive 2-3-day workshop focused on the NIH peer review processes and development of mentees’ specific aims pages, followed by online sessions for group review of mentees’ proposals in progress, and a one-day wrap-up that includes a practice study section on mentees’ advanced drafts by senior researchers. The program is conducted over 3-4 months. The Coach or Advisor helps to mentor those in the Program. More detailed descriptions of each model and data on the efficacy of each, including numbers of proposals submitted, reviewed, resubmitted, and funded have begun to accrue.

In addition to grantsmanship coaching groups, NRMN is addressing the unique needs of investigators who have applied for federal research grants or career development awards but have not yet been successful in obtaining funding. Through “Shark Tank” consultations, NRMN expert grant coaches called “Sharks” provide investigators with intensive and tailored coaching based on the investigators NIH Summary Statement and professional trajectory as detailed in the curriculum vitae and NIH biosketch. These sections are essential areas in the grant review process. The advisors/coaches also provide feedback to mentees with the goal of helping them strategize and fill specific gaps for grant success; e.g. if an investigator received a poor impact/priority score during the study section review,. NRMN coaches/sharks draw upon their own experience to advise on strengthening the research methods, preliminary data, and/or publications.

#### Building capacity for training beyond NRMN

##### Facilitator training

To expand and enhance training beyond NRMN, the program offers train-the-trainer workshops to empower others to implement mentor and mentee trainings [37]. These include: implementation workshops aimed at helping participants plan for ways to bring mentor and mentee training to their program; facilitator training workshops in which participants experience mentor and/or mentee training and then learn how to implement training at their home institutions; and facilitator training for those interested in leading mentor training in an online venue. To date, 27 national workshops have trained 824 participants. Because of these workshops, attendees have reported increased confidence in their ability to facilitate research mentor and mentee training and to implement research mentor and mentee training at their respective institutions. Follow- up surveys allow tracking of implementation events. Similar approaches are being discussed for other NRMN training initiatives.

##### NRMN master facilitators

In order to build continued capacity for research mentor and mentee training, NRMN has recruited 41 individuals from 20 institutions across 14 states in the US who are serving as NRMN Master Facilitators. These individuals have experience implementing mentor training, mentee training, and facilitator training workshops and a passion for improving mentoring relationships for diverse groups. The Master Facilitators participate in annual meetings to share resources and experiences, and implement trainings across the country. This community of Master Facilitators continues to share experiences, resources, and training assessment data through monthly newsletters, quarterly conference calls, and use of the MyGroup feature of MyNRMN.

##### Establishment of an NRMN Institute for Advanced Coaching in Grantsmanship

A critical mass of established investigators nationwide serve as coaches for underrepresented mentees for the mentored intensive grant writing programs. Coaches’ institutes have been established for implementation alongside the Coaching Groups for Grant Writing and Professional Development Programs. The coaches (designated coaches-in-training) for each program are recruited concurrently with the trainees, and the coaches receive “hands-on” and “real time” experience working with the program directors and the trainees during each trainee cohort. The program directors determined that two types of coaches must be involved in the NRMN programs. The first group is the grant writing and professional development coaches. These coaches must be experienced faculty members who have a history of federal funding and/or serving on grant review committees. After they complete training in the NRMN Coaching Group models, these individuals implement and lead participants in a Coaching Group as described above.

The second group of coaches are content experts, with a focused role of ensuring the Coaching Group participants’ grant proposals are both scientifically sound and innovative. The content coaches include the following subcategories: 1) Scientific/Methodology Consultant – typically a 1–2 h specific phone consultation with a junior investigator about scientific questions, approach, or methodology; 2) Scientific/Methodology Coach – individuals who provide scientific discussions and feedback to postdoctoral trainees and junior faculty over a more extended period during preparation of a proposal. These scientific coaches complement the writing and design guidance provided by the NRMN grant writing programs. They provide feedback on research design, approach and drafts of proposal sections over a 2–3 month period – estimated total time 3–10 h, and 3) Proposal Reviewer – individuals who receive a complete or nearly complete draft of a proposal (usually NIH K or R award) and are requested to provide feedback using a standard NIH-reviewer format. These same individuals participate in virtual ‘mock study sections’ led by grant writing program leaders. Proposals are assigned to reviewers according to their scientific and methodological expertise.

#### Goal 3. Refer: Increase access to research resources and career development

All career stages of users on NRMN have the opportunity to benefit from career development programs and activities. For example, in partnership with the Association of American Medical Colleges (AAMC) Graduate Research, Education, and Training (GREAT) group, NRMN offers a series of career development webinars focusing on the graduate school process. NRMN collaborates with partner iBiology on #iBiohangouts to promote and live tweet conversations on Google Hangout. NRMN and iBiology offer a series of discussion panels on topics that revolve around the challenges that diverse scientists face professionally such as the importance of being culturally responsive within mentoring relationships, and typical considerations when negotiating an offer for an academic position. Additional topics range the spectrum from “getting the most out of your conference time” to “developing scientific networks to improve education”. All activities are archived on a NRMN YouTube page. NRMN is also implementing a health disparities learning collaboratory, using a combination of in person, and online social networks, and existing mentor resources at scholars’ home institutions. Such a collaboratory could address the differential access to support, mentoring, and resource infrastructure at each institution. By leveraging networks within the Research Centers in Minority Institutions (RCMI), Clinical and Translational Science Award (CTSA) Program Hubs, and the Mountain West IDeA Clinical and Translational Research –Infrastructure Network (MW CTR-IN), NRMN provides opportunities for virtual engagement while supplying health equity and professional development resources to the target communities, many of who participate in the NRMN Regional Training. The collaboratory offers access to professional development activities including career coaching, lectures, discussions, health equity short courses, and social networking, using functions such as MyNRMN.

#### Goal 4: Promote: Promoting the value of mentoring

To support a cultural transformation in the way mentoring is both conceptualized and valued, NRMN is working with higher education institutions, professional/disciplinary societies and organizations to promote mentoring programs and resources and their importance to faculty development. NRMN has established an NRMN Ambassador Program in which individuals who are committed to the NRMN mission share information about the program at various venues. The types of NRMN Ambassadors include: mentor ambassador, mentee ambassador, NRMN team/staff ambassador, organization ambassador, college-university ambassador, program ambassador, and partner ambassador. Ambassadors represent NRMN through e-mail recruitment campaigns, dissemination of printed materials, website cross-post, conference booth collaboration, social media platform engagement, hosting webinars and/or information sessions, sharing information about NRMN at meetings, conferences, and events attended.

#### Mentor certification

NRMN recognizes that institutions need to value the work mentors do, if real cultural change is to happen. One way to recognize the effort put into mentoring is to create a recognition system for it. Therefore, NRMN has established an NRMN Mentor Certification program through which existing NRMN Mentors can receive certification for their experience and involvement as a mentor within the network. Mentors whose applications are approved by the NRMN will receive a certificate endorsed by NRMN, recognizing their level of experience and skills as a mentor. The three types of Mentor Certifications are as follows:

- **NRMN Mentor**- a mentor who has participated in at least one virtual mentoring cycle as a mentor with a mentee in the Guided Virtual Mentoring Program. This Certificate is also applicable to individuals who have signed up as NRMN mentors and/or served as grant writing coaches through one cycle of an NRMN grantsmanship program
- **NRMN Certified Mentor-** an NRMNet registered mentor who has served as a mentor for at least three different mentees and who has participated in NRMN mentor training (or the equivalent) at their home institution, at special sessions held with partner organizations, or at NRMN sponsored workshops.
- **NRMN Master Mentor** - an NRMN Certified Mentor who has documented significant experience and success as a mentor and has served as a mentor in the NRMN Virtual Mentoring Program for at least one cycle.

### Enhancing NRMN programming: supplement awards, pilot awards and partnerships

#### NRMN supplement and pilot grant projects

To complement the networking, mentoring, and professional offerings initially developed and implemented by NRMN, the leadership issued requests for applications for both Supplement Projects and Pilot Projects, to identify, support, and synergize with other innovative models and programs aligned with NRMN's mission.

**Fig. 5:**
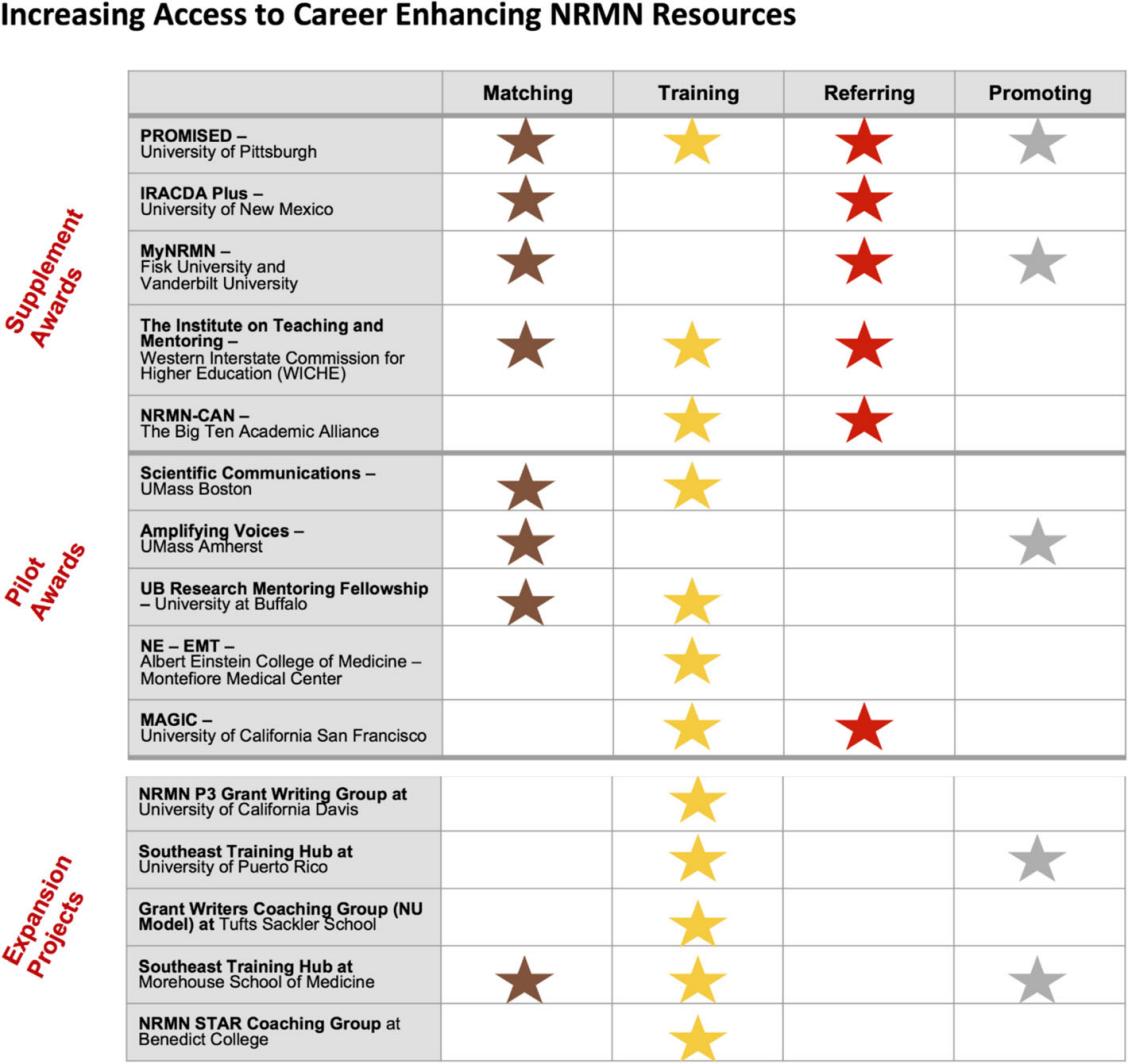
Increasing Access to Career Enhancing NRMN Resources: Supplement Awards, Pilot Awards, and Expansion Projects

The intent was that each supplement or project would coordinate efforts and activities with one or more of the NRMN implementation Cores, and collect relevant metrics and hallmarks of success consistent with the NRMN Evaluation Plan. Summaries of the five NRMN Supplement and five Pilot Projects awarded after both scientific and programmatic review are located on NRMNet. These projects “extend the reach” of NRMN to broader constituencies and regions, fill gaps in NRMN offerings, and build on prior evidence based models of successful professional development and/or mentor/mentee training (Fig. 5 outlines NRMN Supplement Awards, Pilot Awards, and Expansion Projects and their goals). Results from these models will be disseminated and contribute to the evidence base of the future; value-added programming will be integrated into the NRMN implementation cores.

#### NRMN regional training and expansions

NRMN is also expanding its impact in regional training through which scholars in a single geographical region have access to integrated program offerings from across NRMN. Examples of regional trainings included Morehouse School of Medicine for participants in the southeastern United States and the University of Puerto Rico, focusing on three different audiences. Faculty, postdoctoral trainees, and researchers considered as NIH new investigators participate as mentees/trainees in NRMN’s intensive coaching for grantsmanship, which provide a structured group sequence focused on teaching trainees rhetorical patterns and effective writing techniques for NIH-style proposals. Senior faculty with previous experience in developing and securing NIH funds participate as coaches to assist in the training and to learn the model for themselves and their institutions. In addition, institutional officials who occupy leadership positions with access to high-value resources and are able to navigate complex systems and authority to take effective actions, participate in a planning workshop that provides a framework to support the consolidation of mentoring and training activities across disparate programs. This workshop design was based on the structure of the Morehouse School of Medicine Mentoring Academy that provides institutional leaders with a framework to support the consolidation of mentoring and training activities across disparate programs and helps build a critical level of biomedical research capability, productivity and training. The approach uses multidisciplinary research teams and multiple mentors.

## Concluding remarks

NRMN leaders, and all of those working towards its success, are committed to improving research mentorship and career preparation and addressing the challenges of workforce diversity. NRMN has developed and implemented a multifaceted portfolio of programs to enhance national efforts to increase the size, quality, diversity, and research productivity of the biomedical workforce. The Network’s national visibility is wide and its participant reach is growing. The goals of these programs are to: 1) match and link mentees across career stages to mentors and coaches; 2) train mentors, coaches, and mentees to more effectively navigate and maximize their relationships; 3) refer mentees to career and research resources; and 4) promote the value of career mentoring. We look forward to evaluating and disseminating our experiences and best practices. Our strategy will be to leverage “local” and partner resources with NRMN services and expertise, towards the goals of sustainability, scalability, and expanded reach.

## Abbreviations

AAMC: Association of American Medical Colleges
ASPET: American society for pharmacology and experimental therapeutics
BUILD: Building infrastructure leading to diversity
CEC: Coordination and evaluation center
CTSA: Clinical and translational sciences award
DPC: Diversity program consortium
GREAT: Graduate research, education, and training
IRACDA: Institutional research and academic career development awards
MAGIC: Mentoring advisory group in California
MW-CTR-IN: Mountain West IDeA clinical and translational research – infrastructure network
NE-EMT: Northeast evidence-based mentor training consortium
NIGMS: National institute of general medical sciences
NIH: National institutes of health
NRMN: National research mentoring network
NRMN-CAN: NRMN – committee on institutional cooperation academic network
OPM: Optimizing the practice of mentoring
PROMISED: Professional mentoring skills enhanced diversity
RCMI: Research centers in minority institutions
SETH: South Eastern training hub
UW CIRTL: University of Wisconsin center for the integration of research, teaching, and learning

## References

[1] Bland CJ, Taylor AL, Shollen SL, Weber-Main AM, Mulcahy PA (2009). Faculty success through mentoring: a guide for mentors, mentees, and leaders.

[2] Cho CS, Ramanan RA, Feldman MD (2011). Defining the ideal qualities of mentorship: a qualitative analysis of the characteristics of outstanding mentors. Am J Med.

[3] Feldman MD, Arean PA, Marshall SJ, Lovett M, O’Sullivan P. Does mentoring matter: results from a survey of faculty mentees at a large health sciences university. Med Educ Online. 2010;15 10.3402/meo.v15i0.5063 PMC2860862 20431710

[4] Garman KA, Wingard DL, Reznik V (2001). Development of junior Faculty’s self-efficacy: outcomes of a National Center of leadership in academic medicine. Acad Med.

[5] Palepu A, Friedman RH, Barnett RC, Carr PL, Ash AS, Szalacha L (1998). Junior faculty members’ mentoring relationships and their professional development in US medical schools. Acad Med.

[6] Ragins BR, Kram KE (2007). The handbook of mentoring at work: theory, research, and practice.

[7] Ramanan RA, Phillips RS, Davis RB, Silen W, Reede JY (2002). Mentoring in medicine: keys to satisfaction. Am J Med.

[8] Sambunjak D, Straus SE, Marušić A (2006). Mentoring in academic medicine: a systematic review. JAMA.

[9] Shea JA, Stern DT, Klotman PE, Clayton CP, O’Hara JL, Feldman MD (2011). Career development of physician scientists: a survey of leaders in academic medicine. Am J Med.

[10] Steiner JF, Curtis P, Lanphear BP, KO V, Main DS (2004). Assessing the role of influential mentors in the research development of primary care fellows. Acad Med.

[11] Fleming M, Burnham EL, Huskins WC (2012). Mentoring translational science investigators. JAMA.

[12] McGee R, Keller JL (2007). Identifying future scientists: predicting persistence into research training. CBE-Life Sci Educ.

[13] Beech BM, Calles-Escandon J, Hairston KG, Langdon MSE, Latham-Sadler BA, Bell RA (2013). Mentoring programs for underrepresented minority faculty in academic medical centers: a systematic review of the literature. Acad Med.

[14] Thomas DA (2001). **The truth about mentoring minoritie**s. Race matters Harv Bus Rev.

[15] Helm EG, Prieto DO, Parker JE, Russell MC (2000). Minority medical school faculty. J Natl Med Assoc.

[16] Morzinski JA, Fisher JC (2002). A nationwide study of the influence of faculty development programs on colleague relationships. Acad Med.

[17] Ginther DK, Schaffer WT, Schnell J, Masimore B, Liu F, Haak LL (2011). Race, ethnicity, and NIH research awards. Science.

[18] Tabak LA, Collins FS (2011). Weaving a richer tapestry in biomedical science. Science.

[19] Valantine HA, Collins FS (2015). National Institutes of Health addresses the science of diversity. Proc Natl Acad Sci.

[20] National Institutes of Health: Notice of NIH’s Interest in Diversity (NOT-OD-15-053). http://grants.nih.gov/grants/guide/notice-files/NOT-OD-15-053.html. Accessed 2 October 2016.

[21] National Institute for Health Research (NIHR) UK. https://www.nihr.ac.uk/about-us/our-purpose/. Accessed 20 September 2017.

[22] Canadian Institutes of Health Research (CIHR). http://www.cihr-irsc.gc.ca/e/43630.html. Accessed 2 October 2016.

[23] Australian Institute of Health and Welfare (AIHW). http://www.aihw.gov.au/publication-detail/?id=60129549114. Accessed 2 Oct 2016.

[24] Pfund C, Byars-Winston A, Branchaw J, Hurtado S, Eagan K (2016). D**efining attributes and metrics of effective research mentoring relationships**. AIDS Behav.

[25] McGee R (2016). Biomedical workforce diversity: the context for mentoring to develop talents and foster success within the “pipeline**”**. AIDS Behav.

[26] Guererro LR, Ho J, Christie C, Harwood E, Pfund C, Seeman T, McCreath H, Wallace SP. Using collaborative approaches with a multi-method, multi-site, multi-target intervention: evaluating the National Research Mentoring Network. BMC Proceedings 2017, Vol 11 Suppl 12 [S16 this supplement]. 10.1186/s12919-017-0085-6 PMC5773874 29375657

[27] Handelsman J, Pfund C, Miller Lauffer S (2005). Pribbenow, CM. Entering mentoring: a seminar to train a new generation of scientists.

[28] Pfund C, Branchaw J, Handelsman J (2014). Entering Mentoring.

[29] Pfund C, Pribbenow CM, Branchaw J, Lauffer SM, Handelsman J (2006). The merits of training mentors. Science.

[30] Pfund C, House SC, Asquith P, Fleming MF, Buhr KA, Burnham EL (2014). Training mentors of clinical and translational research scholars: a randomized controlled trial. Acad Med.

[31] Branchaw JL, Pfund C, Rediske R (2011). Entering research: Workshops for Students Beginning Research in Science.

[32] Balster N, Pfund C, Rediske R, Branchaw J (2010). Entering research: a course that creates community and structure for beginning undergraduate researchers in the STEM disciplines. CBE-Life Sci Educ.

[33] Nivet MA, Taylor VS, Butts GC, Strelnick AH, Herbert-Carter J, Fry-Johnson YW (2008). Diversity in academic medicine no. 1 case for minority faculty development today. Mt Sinai J Med J Transl Pers Med.

[34] Fleming M, House MS, Shewakramani MV, Yu L, Garbutt J, McGee R (2013). The mentoring competency assessment: validation of a new instrument to evaluate skills of research mentors. Acad Med J.

[35] McDaniels M, Pfund C, Barnicle K (2016). Creating dynamic learning communities in synchronous online courses: one approach from the Center for the Integration of research, teaching and learning (CIRTL). Online Learn J.

[36] Thakore BK, Naffziger-Hirsch ME, Richardson JL, Williams SN, McGee R (2014). The academy for future science faculty: randomized controlled trial of theory-driven coaching to shape development and diversity of early-career scientists. BMC Med Educ.

[37] Pfund C, Spencer KC, Asquith P, Miller S, Sorkness CA, House SC (2015). Building National Capacity for research mentor training: an evidence-based approach to training the trainers. CBE-Life Sci Educ.

